# Metal Accumulation and DNA Damage in *Oreochromis niloticus* and *Clarias gariepinus* After Chronic Exposure to Discharges of the Batts Drain: Potential Risk to Human Health

**DOI:** 10.1007/s00128-022-03512-8

**Published:** 2022-04-13

**Authors:** Moussa Attia Moussa, Hanan Ramadan H. Mohamed, Amr Adel Abdel-Khalek

**Affiliations:** Zoology Department, Faculty of Science, Cairo University, Cairo, Egypt

**Keywords:** Metal accumulation, Genotoxicity, Human hazard index, The Batts drain, *O. niloticus*, *C. gariepinus*

## Abstract

The present work showed the impact of long-term exposure to the Batts drain’s discharges on *O. niloticus* and *C. gariepinus* fish species. The accumulation level of Cu, Zn, Fe, Cd, Pb and Al in five vital tissues was markedly elevated near the Batts drain (site2) compared to the samples from the reference site (site 1). At the same site, *C. gariepinus* displayed the highest accumulation capacities when compared to *O. niloticus*. Based on the metal pollution index, livers and kidneys, followed by gills, showed the maximum overall metal load. The degree of DNA damage (assessed by comet and diphenylamine assays) was relative to the accumulated metals in tissues with species and site specification. The values of the hazard index for human consumption showed that the studied metals were within safe values at normal consumption rates. While harmful health consequences were observed at the habitual consumption level at site 2.

Water pollution is a severe problem due to the growing anthropogenic activities near different aquatic bodies (El Agawany et al. 2021). Increased industrial, agricultural and commercial chemical discharges into water causes a variety of ecological changes in aquatic environments. Despite recent environmental protection legislation, uncontrolled wastewater combined with inadequate water management has degraded many Egyptian lakes (Abdel-Khalek et al. 2020). Lake Qaroun lies about 80 km southwest of Cairo, in the Fayoum depression on Egypt's western desert border. It is a confined basin with a 45-km length, 5.7-km width, 4.2-average depth and 243 km^2^ surface area (Redwan and Elhaddad 2017). The lake receives most of the agricultural drainage water via two main drains, the Batts and the Wadi, in addition to smaller drains that discharge small amounts of the drainage water into the lake. The Batts is the significant drain that accumulates agricultural wastes from the Fayoum province's eastern and northeastern regions and discharges those wastes directly into Lake Qaroun. This huge drain collects around 450 million m^3^/year of agricultural and domestic wastes (Elwasify et al. 2021). Therefore, Lake Qaroun faces several environmental problems that badly affect its living organisms due to receiving extraordinary quantities of untreated water discharges carrying several aquatic contaminants. Anthropogenic metals are a fascinating category of aquatic pollutants due to their adverse effects on ecosystem constancy, high accumulative potency in various tissues and their long-term persistence in water (Abdel-Khalek et al. 2020). Among toxic metals, cadmium (Cd), lead (Pb) and aluminium (Al) are non-essential metals that may induce toxicity even at low concentrations. In contrast, essential metals such as copper (Cu), iron (Fe) and zinc (Zn) are trace elements that have significant roles in many biological processes and become toxic if their concentration exceeds a certain threshold (Zaghloul et al. 2011; Elwasify et al. 2021). Metals accumulate in the fish body directly via the contact of gills and skin with the external environment or through the ingestion of metal-contaminated food particles (Tunçsoy et al. 2017). As long as metals' absorption and accumulation rates exceed the excretion and detoxification rates, the metallic toxicological impacts on different tissues are expected to increase (Xu et al. 2021). Studies that have been conducted on fish that chronically inhabit contaminated areas are more realizable than those conducted in controlled laboratory conditions (Hamilton et al. 2016). Abdel-Khalek et al. (2018) reported that the data obtained from field studies have significant ecological relevance and helps in better understanding the actual interactions between metals and their effects on wild fish. Moreover, it is hard to evaluate fish health using routine chemical analysis because aquatic organisms can react differently to the same contaminant. Therefore, fish are frequently used as bio-indicators in many toxicological studies (Paul et al. 2019). Nile tilapia (*O. niloticus*) and African catfish (*C. gariepinus*) have great economic importance, broad zoogeographic distribution and tolerate several environmental pollutants. Therefore, they are usually used to reflect the water quality and the impacts of deteriorating environmental conditions. Accordingly, using integrated biomarkers to monitor environmental stressors and their impacts on aquatic biota has become a valuable and reliable tool (Abdel-Khalek 2015). Various metals are considered genotoxic pollutants due to their destructive impacts on the DNA integrity of aquatic organisms (Kontaş and Bostancı 2020). Therefore, environmental genotoxicity screening gives early warning signs of the negative long-term impacts on the exposed organisms. Several techniques for assessing genotoxicity, including the alkaline comet assay, single-cell gel electrophoresis (SCGE) and the diphenylamine assay, are widely used. Researchers prefer the comet assay due to its high sensitivity, low cost, simplicity and quick capability of detecting DNA damage (Pellegri et al. 2020). The diphenylamine assay was also used in the present study as a complementary assay with the alkaline comet assay to quantify the overall fragmented DNA and determine the severity of genotoxic damage. A comprehensive investigation of the bioaccumulation levels of potentially hazardous metals in various tissues of fish is an indicative biomarker for assessing the expected health hazards of fish consumers. The metal pollution index (MPI) and the hazard index (HI) are investigated indices that are used to evaluate the impact of metal intake through fish consumption on human health (Ali and Khan 2018). Therefore, the present study aimed to (1) evaluate the impact of the Batts drain on two economically valuable fish species (*O. niloticus* and *C. gariepinus*) inhabiting the same habitat based on the accumulation potency of the studied metals and the integrity of DNA in tissues; and (2) assess the human health hazards related to fish consumption from those studied sites.

## Materials and Methods

**Fig. 1:**
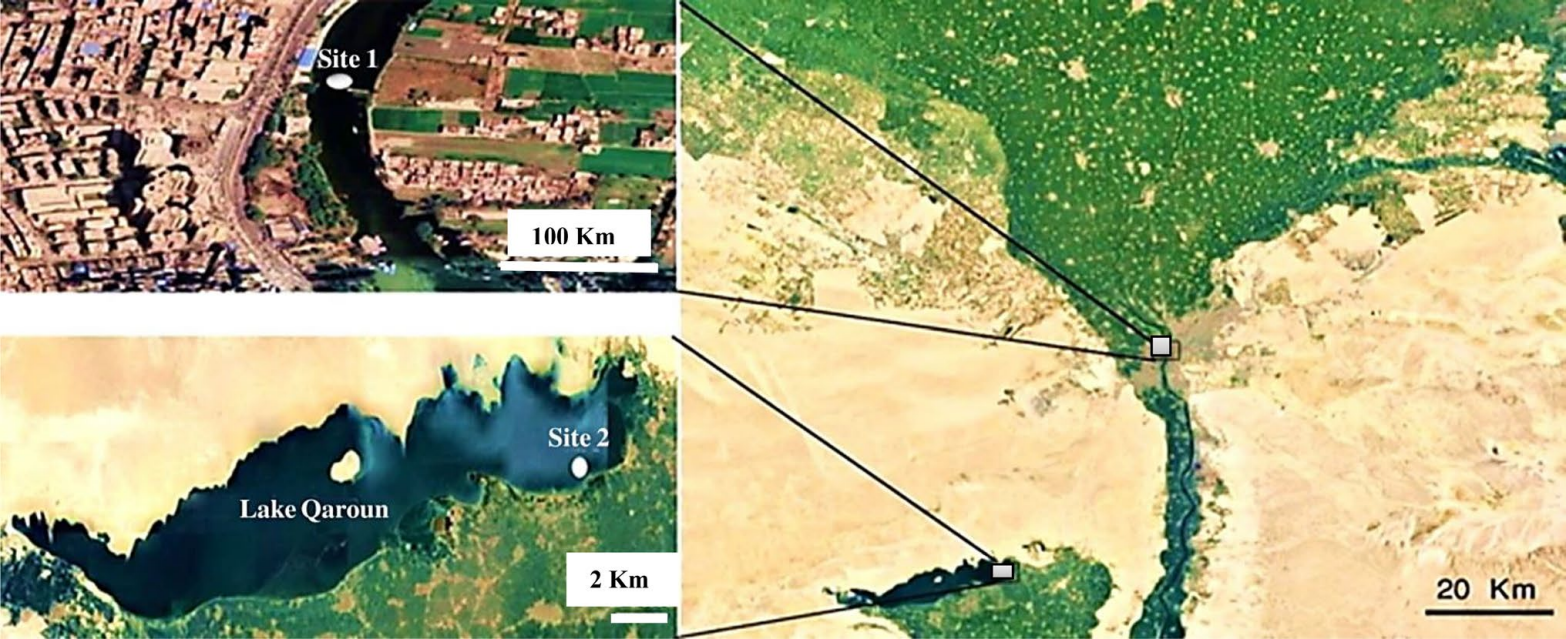
The selected sampling sites, site 1 represents the reference site (River Nile at South of Giza governorate) and site 2 represents the polluted site (at the discharge inlet of the Batts drain)

Sites description as shown in Fig. 1. Site 1 (River Nile at South of Giza governorate), represents the reference site due to the distance from this site from any source of contamination and waste discharge. In addition, all recorded metals in water and sediment (unpublished data) were below the guideline values recommended by MacDonald et al. (2000) and MWRI (2013). GPS: 30° 00ʹ 02.034ʺ N and 31° 12ʹ 55.7532ʺ E. While, site 2 (at the discharge inlet of the Batts drain) as most anthropogenic activities are located at the eastern section of Lake Qaroun and the main agricultural drains from the Batts drain. The Batts drain spontaneously releases its contents into the eastern part of Lake Qaroun, so site 2 was chosen in the immediate vicinity of the Batts’ discharge point. GPS: 29° 28ʹ58.98ʺ N and 30° 49ʹ08.02ʺ E.

*O. niloticus* and *C. gariepinus* were collected from the studied sites during the summer season, 2019 (highest evaporation and pollution rates), with the help of expert fishermen. Thirty-six adult, healthy and male fish (9 fish/species/site) were selected for this study. The body weights and lengths were 195.51 ± 7.46 g; 19.95 ± 0.36 cm for *O. niloticus* and 353.67 ± 24.4 g; 37.91 ± 1.02 cm for *C. gariepinus*. Fish samples were delivered to the ecology laboratory, Zoology Department, Faculty of Science, Cairo University, in large plastic containers with two portable oxygen pumps for each one. After decapitation, the dissection of the fish was done, then liver, kidney, gills, skin and muscle tissues were obtained. The Institutional Committee on Animal Care and Use (IACUC) of Cairo University, Faculty of Science, Cairo, Egypt reviewed and accredited this study with the accreditation no. CU-I-F-39-19.

The concentrations of Cu, Zn, Fe, Cd, Pb and Al metals were determined in five selected tissues of both species using Inductively Coupled Argon Plasma, ICAP 6500 Duo Thermo Scientific, England. For instrumental standardization, a 1000 mg/L multi-element certified standard solution (Merck, Germany) was utilized as a stock solution. The limits of detection in ppm were < 0.002 for Cu, < 0.001 for Al, < 0.0005 for Cd, < 0.005 for Zn, Pb and Fe. The acid digestion method was conducted as fully described by Neugebauer et al. (2000). The tissue samples were dried in 20 mL crucibles for eight hours at 80°C. The samples were acid digested using concentrated hydrochloric acid then placed on a heat plate (100°C) until getting clear solutions. The clear solutions were diluted to a known volume in a 25-ml volumetric flask using de-ionized water. Metal concentrations were expressed as mg/kg dry weight. Blank samples (prepared in just the same way as digested samples only without the sample) were prepared together for each sample set to adjust background absorption. Standard reference material (Lake Superior fish 1946 NIST, National Institute of Standards and Technology, USA) was used to verify the analytical method. The recovery range was between 97 and 103 percent. After every four samples, a standard solution with known metal concentrations was measured to verify the measurement accuracy.

Using the formula of Usero et al. (1997), this index was determined to evaluate a whole load of metals in the studied tissues of both fish species: MPI = (M1 × M2 × M3 × …..Mn)1/n, where Mn is the concentration of metal (mg/kg dry wt.) in a specific tissue and n, represents the number of the studied metals.

The alkaline (pH > 13) comet assay was conducted according to the method described by Tice et al. (2000) and Recio et al. (2010). Small pieces of liver and gills were placed in 1 mL cold Hank's balanced salt solution (HBSS; Ca^++^ & Mg^++^ free) containing 20 mM EDTA and 10% DMSO. Then, A 10 µL aliquot of cell suspension was mixed with 80 µL of 0.5% low melting point agarose (Sigma) and diffused on a fully frosted slide pre-dipped in normal melting agarose (1%). After solidification, the slides were positioned in cold lysis buffer (2.5 M NaCl, 100 mM EDTA and 10 mM Tris, pH 10, with freshly added 10% DMSO and 1% Triton X-100) for 24 h at 4°C away from any source of light. Subsequently, the slides were placed in an alkaline buffer (300 mM NaOH and 1 mM EDTA, pH > 13) for 20 min. The unwinded DNA samples were electrophoresed for 20 min at 300 mA and 25 V (0.90 V⁄cm). After electrophoresis, slides were immersed in an excess amount of 0.4 M Trizma base (pH 7.5) to neutralize the alkali. Then slides were fixed in 100% cold ethanol, air-dried and finally stored at room temperature in a desiccator until they were scored. Slides were stained with ethidium bromide (2 µg⁄mL) then, images were captured at × 400 magnification by a fluorescence microscope (Optika B 353LD2 LED trinocular fluorescence microscope). DNA damage was measured as the amount of fluorescence in the comet tail (% DNA in comet tail) using TriTek CometScoreTM (Freeware image-analysis software v1.5). In the present study, 250 cells/tissue/species/site were estimated.

Both tissues (25 mg) were lysed in 0.8 mL of cold lysis buffer (10 mM Tris–HCl; pH 8, 1 mM EDTA, 0.2% Triton X-100). The resulted homogenates were centrifugated at 10.000 r.p.m. for 10 min at 4°C to separate the intact DNA (in the pellet) from fragmented DNA (in the supernatant). The resulting pellet and supernatant were mixed with 1.5 mL Tris–EDTA buffer (1 mM EDTA, 10 mM Tris, pH 8.0) containing 10% trichloroacetic acid (TCA) in separate tubes. After 10 min of incubation at room temperature, each test tube was centrifuged at 3000 r.p.m. (at 4°C) for 15 min, then the supernatant was removed. The precipitates from each tube were mixed with 0.7 mL of 5% TCA, boiled for 15 min, cooled and then centrifuged at 2000 r.p.m. for 15 min. one ml of the diphenylamine reagent (1.5 g diphenylamine in 100 mL of acetic acid, 1.5 mL H_2_SO_4_ and 16 μg/mL acetaldehyde) was added to 0.5 mL supernatant of each tube. After overnight incubation, incubated the resulting blue color was measured at 600 nm against diphenylamine solution as a blank solution. The quantity of damaged DNA was expressed as a percentage of total DNA the equation of Gercel-Taylor (2001).

DNA fragmentation = Absorbance of fragmented DNA (represented by supernatant) / [Absorbence of fragmented DNA (represented by supernatant) + Absorbance of intact DNA (represented by pellet)] × 100.

The mean daily ingestion of a particular chemical over a lifetime was determined using the equation stated by USEPA (2000) in eaten (muscles and skin) tissues of studied fish. ADD (mg/kg/day) = (C × IR × EF × ED/((BW × AT) where C is the concentration of each metal (average concentration in mg/kg was used for each metal). IR is the mean ingestion rate (0.0312 kg/day for normal consumers and 0.1424 kg/day for habitual ones). EF equals 365 days/year, representing the exposure frequency. ED represents the exposure duration over a lifetime, which equals 70 years. BW is the average body weight (70 kg for normal adults); AT is the average time (70 years × 365 days/year). The hazard index (evaluates the impact health effects from intake of specific metal in food) was determined from the following equation; Hazard Index (HI) = ADD/ oral RfD where oral RfD represents the oral reference dose (the maximum dose at which no adverse health effects are expected) of a particular metal (mg/kg/day), which is based on USEPA (2012) and Antoine et al. (2017). The values of oral RfD are 0.04, 0.3, 0.7, 0.003, 0.001 and 1.0 mg/kg/day for Cu, Zn, Fe, Pb, Cd and Al respectively. Based on the HI values, HI < 1 means that there are no adverse effects while HI ≥ 1 means that risk effects may occur.

The results were expressed as a mean ± standard error. The student's t-test was statistically used to analyze data (*p* < 0.05) to estimate differences between studied fish species at the same site and evaluate the difference between the same species at different sites. Data were analyzed using Statistical Processor Systems Support, SPSS software, version 25.0, IBM, Chicago, IL, USA.

## Result

The mean concentration of six accumulated metals in the liver, kidney, gills, skin and muscle tissues of both studied species from two sites was summarized in Table 1. The average metal levels in different tissues of both species were significantly (*p* < 0.05) higher at site 2 than at site 1. *C. gariepinus* revealed a significantly (*p* < 0.05) higher accumulation of most studied metals in various tissues than *O. niloticus*. *C. gariepinus* showed higher Cu content in the liver and gills compared with *O. niloticus* at site 1, while the Cu content in *C. gariepinus* was significantly elevated in all tissues except the muscle compared to *O. niloticus* at site 2. The accmulated Zn in *C. gariepinus* was significantly increased in all studied tissues (except the muscle) compared to *O. niloticus* at site 1. Iron concentrations in the tissues of *C. gariepinus* were markedly elevated in the liver and kidney (site 1) and kidney, gills and muscle (site 2) compared to *O. niloticus* at the same site. The hepatic Pb content of *C. gariepinus* was elevated at site 1, while at site 2, Pb contents in the liver, gills and muscles were increased compared to *O. niloticus*. The accmulated Cd in *C. gariepinus* was significantly increased in all studied tissues (except the muscle at site 1 and the skin at site 2) compared to *O. niloticus*. The accumulated Al of *C. gariepinus* was significantly increased in the kidney and gills at site 1 and in all tissues at site 2. The accumulation patterns of metals showed a high ability of the liver, kidney and gills to accumulate more metals than skin and muscular tissues.

**Table 1:** Metal accumulation (mg/kg dry wt.) in five tissues of *O. niloticus* and *C. gariepinus* collected from the studied sites

	Site 1 (Reference site)		Site 2 (The Batts site)	
	*O. niloticus*	*C. gariepinus*	*O. niloticus*	*C. gariepinus*
Copper				
Liver	18.36 ± 3.23B	28.11 ± 2.39B*	86.27 ± 2.61A	123.51 ± 1.25A*
Kidneys	18.87 ± 2.16B	26.2 ± 4.6B	80.71 ± 5.21A	98.74 ± 4.3A*
Gills	11.97 ± 0.71B	18.73 ± 1.49B*	28.84 ± 3.45A	45.22 ± 4.05A*
Skin	7.48 ± 1.12B	9.57 ± 1.89B	13.05 ± 1.49A	20.21 ± 2.25A*
Muscles	8.37 ± 0.45B	10.27 ± 0.96B	14.11 ± 2.25A	17.44 ± 0.26A
Zinc				
Liver	67.2 ± 0.03B	105.86 ± 5.18B*	123.76 ± 11.62A	150.59 ± 18.57A
Kidneys	67.98 ± 8.56B	141.71 ± 1.95B*	229.02 ± 5.05A	278.45 ± 23.7A
Gills	24.25 ± 2.92B	50.15 ± 5.96B*	120.45 ± 1.99A	128.48 ± 5.37A
Skin	119.65 ± 11.13B	182.19 ± 3.8A*	181.89 ± 17.61A	510.06 ± 207.7A
Muscles	38.85 ± 5.52B	48.54 ± 3.26B	69.60 ± 4.40A	70.26 ± 7.62A
Iron				
Liver	86.66 ± 3.7B	139.2 ± 15.4B*	267.54 ± 4.4A	377.48 ± 53.58A
Kidneys	47.32 ± 4.32B	64.33 ± 3.7B*	189.75 ± 31.34A	307.82 ± 32.35A*
Gills	64.14 ± 5.71B	72.832 ± 7.47B	222.06 ± 14.37A	341.12 ± 34.9A*
Skin	23.75 ± 3.07B	23.9 ± 4.76B	182.64 ± 28.31A	178.59 ± 26.76A
Muscles	15.68 ± 0.57B	15.4 ± 0.32B	164.13 ± 13.00A	24.24 ± 20.31A*
Lead				
Liver	0.137 ± 0.006B	0.51 ± 0.08B*	1.4 ± 0.09A	2.13 ± 0.24A*
Kidneys	0.78 ± 0.01B	0.73 ± 0.06B	2.21 ± 0.12A	2.67 ± 0.20A
Gills	0.44 ± 0.03B	0.53 ± 0.11B	1.43 ± 0.02A	2.28 ± 0.21A*
Skin	0.07 ± 0.02B	0.12 ± 0.02B	0.69 ± 0.076A	0.73 ± 0.02A
Muscles	0.04 ± 0.01B	0.03 ± 0.01B	0.52 ± 0.1A	0.81 ± 0.03A*
Cadmium				
Liver	1.63 ± 0.09B	2.57 ± 0.06B*	3.12 ± 0.22A	7.2 ± 0.49A*
Kidneys	0.98 ± 0.04B	1.45 ± 0.093B*	5.61 ± 0.57A	8.15 ± 0.45A*
Gills	0.81 ± 0.04B	1.1 ± 0.09B*	1.6 ± 0.17A	2.85 ± 0.2A*
Skin	0.33 ± 0.04B	0.48 ± 0.05B*	0.63 ± 0.09A	0.77 ± 0.07A
Muscles	0.23 ± 0.02 B	0.25 ± 0.03B	1.19 ± 0.07A	2.19 ± 0.31A*
Aluminum				
Liver	11.01 ± 0.987B	17.47 ± 1.5B	54.87 ± 3.55A	162.4 ± 6.34A*
Kidneys	8.28 ± 0.04B	13.27 ± 0.92B*	125.74 ± 2.47A	225.87 ± 31.64A*
Gills	8.20 ± 0.004B	14.16 ± 1.17B*	133.84 ± 1.8A	208.58 ± 11.6A*
Skin	4.12 ± 0.31B	3.36 ± 0.1B	18.67 ± 1.2A	46.57 ± 2.8A*
Muscles	3.2 ± 0.53B	3.6 ± 0.11B	18.05 ± 1.4A	41.1 ± 2.87A*

Data are represented as means of six samples of each species per site ± SE

The capital letters represent significant differences (t-test; *p* < 0.05) between the same species of different sites. Values with the same capital letters for each metal are not significantly different

Symbol asterisk means that values are significantly different (t-test; *p* < 0.05) for different species at the same site

This index exhibited the overall accumulated metals of both selected fish species (Table 2). The values of MPI were higher in the fish of the Batts site than those of the reference site in all studied tissues. Based on the values of this index, hepatic and renal tissues showed the maximum accumulation capacities, followed by gills in both studied fish. While the lowest accumulation rate was observed in the muscular tissues.

**Table 2:** Metal pollution index (MPI) in vital tissues of *O. niloticus* and *C. gariepinus* from studied sites

	Site 1 (Reference site)		Site 2 (The Batts)	
	*O. niloticus*	*C. gariepinus*	*O. niloticus*	*C. gariepinus*
Liver	8.004	14.549	29.687	50.946
kidneys	8.526	12.235	41.972	58.863
Gills	6.155	9.091	24.862	37.283
Skin	3.556	4.478	12.333	19.076
Muscle	2.305	2.433	11.029	16.479

**Fig. 2:**
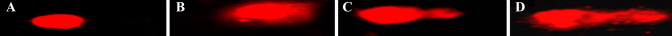
Comet assay in liver and gills of *O. niloticus* and *C. gariepinus* showing different DNA levels of damage. The magnification power was × 400. **A** Undamaged cell; **B**–**D** different levels of DNA damages

Similarly, the level of genomic DNA fragmentation in *O. niloticus* and *C. gariepinus* which chronically inhabit site 2 was compared to that of the reference site. The smeared appearance of damaged DNA in the cells of the liver and gills was recorded and compared in terms of % of DNA in the tail as represented in Fig. 2. The obtained results (Table 3) showed the same trend as in the DPA technique with a slight difference. The highest values of DNA damage in both tissues were recorded in site 2. The percentage DNA breakage in liver tissue revealed significant (*p* < 0.05) elevation in *C. gariepinus* compared to *O. niloticus* inhabiting the same site. In gills, the % of DNA in the tail showed insignificant differences between different species of the same site.

**Table 3:** The percentage of DNA in the comet tail

	% DNA in tail			
	Site 1 (Reference site)		Site 2 (The Batts)	
	*O. niloticus*	*C. gariepinus*	*O. niloticus*	*C. gariepinus*
Liver	5.563 ± 0.55B	8.618 ± 0.999B*	17.896 ± 1.38A	23.209 ± 1.62A*
Gills	7.763 ± 0.787B	9.363 ± 0.950B	22.433 ± 1.654A	23.238 ± 1.368A

Data are represented as means of 250 cells/tissue/species/site ± SE

The capital letters represent the significant difference (t-test; *p* < 0.05) between the same species of different sites. Values with the same capital letters for each tissue are not significantly different

Symbol asterisk means that values are significantly different (t-test; *p* < 0.05) for different species at the same site

To quantify the % of DNA damage in both fish species, the liver (the main site for detoxification and biotransformation processes) and gills (in continuous exposure to external stressors) were selected. The percentages of DNA damage showed significant variability through different species and sites.

As shown in Table 4, the long exposure to the Batts’ discharges induced DNA damage in the liver and gills as assessed by colorimetric measuring of the damaged DNA level. The total DNA damage in liver tissue revealed significant (*p* < 0.05) elevation in *C. gariepinus* compared to *O. niloticus* at site 1. While at site 2 the total DNA damage was significantly elevated in the gills of *C. gariepinus* at site 2.

**Table 4:** The percentage of whole damaged DNA in livers and gills of *O. niloticus* and *C. gariepinus* collected from studied sites

	Site 1 (Reference site)		Site 2 (The Batts)	
	*O. niloticus*	*C. gariepinus*	*O. niloticus*	*C. gariepinus*
Liver	33.44 ± 1.23B	43.41 ± 1.95B*	60.87 ± 3.46A	61.19 ± 3.6A
Gills	36.33 ± 2.42B	40.33 ± 0.505B	48.41 ± 0.076A	54.24 ± 1.52A*

Data are represented as means of six samples of each species per site ± SE

The capital letters represent significant differences (t-test; *p* < 0.05) between the same species of different sites. Values with the same capital letters for each tissue are not significantly different

Symbol asterisk means that values are significantly different (t-test; *p* < 0.05) for different species at the same site

Hazard index (HI) values for analyzed metals in the muscle and skin (edible tissues) were presented in Table 5. The HI values for edible fish tissues of both species were less than 1.0 at the normal consumption level, while harmful health consequences were observed at the habitual consumption level, especially for Cd and Zn levels at site 2. Although the low HI values for each metal (HI < 1) at a normal consumption rate, the combined cumulative risk effects of all accumulated metals are a danger sign for fish consumer health around the studied sites.

**Table 5:** Hazard index for muscle (HI_m_) and skin (HI_S_) consumption of *O. niloticus* and *C. gariepinus* calculated at mean normal ingestion rate (0.0312 kg/day) and subsistence ingestion rate (0.1424 kg/day)

	Site 1 (reference site)				Site 2 (Batts)			
	*O. niloticus*		*C. gariepinus*		*O. niloticus*		*C. gariepinus*	
	Normal	Habitual	Normal	Habitual	Normal	Habitual	Normal	Habitual
Cu								
HI_m_	0.0932	0.4256	0.1144	0.5223	0.1572	0.7175	0.1943	0.0354
HI_s_	0.0833	0.3804	0.1066	0.4867	0.1454	0.6636	0.2252	0.0411
Zn								
HI_m_	0.0577	0.2634	0.0721	0.3291	0.031	0.4719	0.1043	0.476
HI_s_	0.1777	0.8113	0.2706	1.235	0.270	1.233	0.7578	1.037
Fe								
HI_m_	0.00998	0.0455	0.00980	0.0447	0.1045	0.4769	0.1427	0.6516
HI_s_	0.01512	0.069	0.0152	0.0694	0.1162	0.5307	0.1137	0.5190
Cd								
HI_m_	0.1025	0.4678	0.1114	0.5085	0.5304	2.4208	0.9761	4.4550
HI_s_	0.1470	0.6713	0.2139	0.9764	0.2808	1.2816	0.3432	1.5664
Pb								
HI_m_	0.00594	0.0271	0.00445	0.0203	0.0772	0.3526	0.1203	0.5492
HI_s_	0.0104	0.0474	0.0178	0.0813	0.1025	0.4678	0.1084	0.4950
Al								
HI_m_	0.0014	0.0065	0.0016	0.0073	0.0080	0.0367	0.0183	0.0836
HI_s_	0.0018	0.0083	0.00149	0.0068	0.0083	0.0379	0.020	0.0947
Cumulative values_(m)_	0.2707	1.2359	0.3137	1.4322	0.9083	4.4764	1.556	6.2508
Cumulative values_(S)_	0.4353	1.9877	0.6256	2.8556	0.9232	4.2146	1.5683	3.7532

## Discussion

Health problems induced by chronic exposure to metal pollution cannot be assessed or expected in living organisms using chemical analysis or classical environmental monitoring techniques (Abdel-Khalek et al. 2018). Therefore, using integrated biomarkers and suitable bioindicators is strongly recommended in several toxicological studies to assess the real impacts of environmental pollutants on several health aspects (Javed et al. 2016). In accordance with the current findings, Abdel-Khalek et al. (2020) demonstrated an excessive increase in the concentration of metals in the eastern part of Lake Qaroun in sediments and water samples, with the highest concentration during the summer season. Rajeshkumar et al. (2018) reported that excessive evaporation rates during the high-temperature season are the main cause of elevated pollutant concentrations in different aquatic bodies. The high density of anthropogenic activities close to the eastern section of Lake Qaroun and the tremendous effluents that flow throughout the Batts drain have greatly affected optimal environmental conditions for many fish species. In addition, continuous exposure to unprocessed releases with high metal loads can deteriorate the health status of chronically exposed fish. Monitoring of metal concentrations in water and sediment is important in recognizing the level of pollution in certain aquatic environments but it is difficult to rely on this routine examination to assess the general health condition of fish (Khallaf et al. 2018). Fish absorb metals through three mechanisms: direct contact with the skin, respiratory surfaces and ingestion of contaminated particles (da Mota Araujo et al. 2018). The absorbed metals are subjected to distribution (bloodstream delivery of metals to tissues), biotransformation (chemical alteration of metals to increase their excretion rate) and excretion (a sequence of mechanisms to eliminate metals from the body) processes. When the absorption rate exceeds the excretion rate, the accumulation of metals will increase then negative consequences will be initiated in many vital tissues (Xu et al. 2021). The accumulation rate differs among tissues based on the rate of blood supply and the affinity of tissue to the absorbed metals. The high blood supply and the richness of metal-binding proteins such as metallothioneins (MTs) may explain the elevated metal content in the hepatic and renal tissues of the studied fish. Accumulated metals can prompt gene expression of MTs to immobilize and detoxify those metals. MTs have a cysteine content of 25%–35%; therefore, these binding proteins provide a high number of thiol groups (-SH) that strongly bind different metals forming metal-tetrathiolate clusters (Nagamatsu et al. 2021). The stoichiometric properties of thiol groups in cysteine residues are responsible for their selective and competent binding with different metals (Samuel et al. 2021). Moreover, divalent metal transporter 1 (DMT1) is abundantly expressed in the liver and kidneys to facilitate the transportation process of various divalent cations such as Cd^2+^, Pb^2+^, Zn^2+^, Ni^2+^ and Pt^2+^; therefore, both tissues are more susceptible to metallic damage (Barbier et al. 2005). Gills are the major pathway for many waterborne minerals' uptake due to their role in gas exchange, ionic regulation and high surface area. Gills consist of primary and secondary lamellae with many squamous epithelial cells, chloride cells, mucous and interlamellar cells. Chloride cells are specialized ion-transporting cells and most of their apical membrane is covered by pavement cells (PVC). Only a small part of their apical membrane is in direct contact with the water so PVC may control the apical absorption of ions by altering the small part of the chloride cell region exposed to water (Lall and Kaushik 2021). Chloride cells (ionocytes) have unique transporters or channels (for example sodium–potassium ATPase) on the apical and basolateral membranes to transport ions through chloride cells (Chaudhuri et al. 2021). Prolonged exposure to high metal concentrations can interfere with those transporters and inhibit enzymes that facilitate ion transport through chloride cells. For example, excessive Cu and Al metal uptake via chloride cells can compete with Na^+^ absorption and inhibit Na^+^ K^+^ ATPase activity, leading to a high metal accumulation rate, osmoregulatory disturbances, epithelium damage and respiratory dysfunction (Russell et al. 2019). Abdel-Khalek et al. (2020) reported that the negativity of the gill surfaces is the major cause of high concentrations of positively charged metals. Therefore, excessive and continuous exposure to divalent metals such as Cd^+2^, Zn^+2^ and Pb^+2^ may increase the accumulation rate of these metals in the gills, as confirmed in the present study. On the other hand, the lowest concentration of all studied metals was observed in the skin and muscular tissues of both species. The low metabolic activity and scarcity of both metal transporters and metal-binding proteins may decrease the accumulation capacities of dermal and muscular tissues for all studied metals. This result was consonant with Khallaf et al. (2018), who showed lower muscular content of metals compared to a high accumulation rate in the liver and kidneys. To verify and evaluate the genotoxic damage in both studied species, the level of DNAfragmentation was qualitatively and quantitatively determined in the liver (which represents the detoxification tissue) and gills (which represent the respiratory surfaces that are continuously exposed to xenobiotics). Javed et al. (2016) mentioned that genotoxicity and other biochemical changes can be induced by long-term exposure to different metals in *Channa punctatus.* Shah et al. (2021) showed that lead, chromium and copper can cause genotoxic effects in common grass carp (*Ctenopharyngodon Idella*). Freshwater fish, *Labeo rohita* have been used to investigate DNA fragmentation induced by different concentrations of cadmium chloride (Jindal and Verma 2015). Morovere, Ratn et al. (2018) reported that high accumulation of zinc metals induced DNA damage and other responses in *Channa punctatus*. The initiation of apoptotic DNAdamage was indicated in the present work through comet appearance (a smeared pattern of DNA) and the high percentage of fragmented DNAin the liver and gills collected from site 2. The active biotransformation processes in the hepatic tissues and the structural location of the gills may increase the exposure rate of both tissues to genotoxic metals from their surroundings. Long-term exposure to genotoxic metals can directly affect DNAintegrity by causing physical disruption as DNAmolecules are a suitable substrate for the attachment of metal cations (Kontaş and Bostancı 2020). Metals may also inhibit essential proteins and enzymes involved in DNA-repair pathways (Javed and Usmani 2019).Cells deal with DNAdamage by stimulating potent DNArepair cascades, which require enough time for the expression of key enzymes required for the proofreading process. This time is also important to allow the physical removal of the damaged part of the DNAmolecule in a substrate-dependent manner (Chatterjee and Walker 2017). The high percentage of DNAfragmentation at site 2 indicated that the continuous exposure to several genotoxic metals could impair the expression of the required proofreading enzymes and, consequently, the efficient repair mechanisms. Javed and Usmani (2019) concluded that metals could cause genotoxicity via the generation of reactive oxygen species that attack nucleotides and cause deoxyribose sugar oxidation. The level of DNAdamage was elevated in the *C. gariepinus* compared to *O. niloticus* of the same site. This elevation was in line with the results of the accumulation of metals in fish tissues, which revealed a significant correlation between the accumulated metals and the level of DNAdamage. Fish are one of the vital sources of protein for humans. Therefore, the accumulated pollutants in fish tissues pose health threats to individuals who depend on fish in their meals (Adeogun et al. 2020). High metal accumulation in edible tissues (muscle and skin) could pose a significant health hazard to human health (Pal and Maiti 2018). When metals exceed the normal ranges in the human body, several negative effects will be expected due to the disturbance of many cellular functions and biochemical reactions (Ali and Khan 2018). In the present study, fish's muscle and skin (edible tissues) tissues seemed to accumulate the lowest levels of metals compared to other studied tissues. But, to assess health hazards, many factors other than metal concentrations should be considered, as the duration and frequency of exposure, body weight and oral reference dose (Javed and Usmani 2019). Taking into account all of these factors, the values of HI showed that normal fish consumers are not at risk, but the risk is likely to be greatly increased for fish-dependent consumers, especially at site 2. Inaddition, several metals in their massive form are found in edible tissue, which can extraordinarily harm consumers’ health. As a result, the cumulative risk impact of all metals should not be overlooked. Waseem and Arshad (2016) reported that high metal intake levels could pose severe negative impacts on human health, such as precipitation of phosphate bio-compounds, deformation of the active site of important enzymes and destruction of several macromolecules (protein, lipid and DNA).

The untreated discharges of the Batts drain had a potential health concern as indicated by high metal accumulation and genotoxic damage in exposed fish. The severity of DNA fragmentation was significantly different in the studied fish species and was directly proportional to the level of metal accumulation in their tissues. The values of HI for humans showed that harmful health consequences were expected, especially for habitual fish consumers at site 2. Therefore, water treatment and constant monitoring procedures should be applied at site 2 to overcome the detrimental impacts of anthropogenic discharges.

## References

[CR1] Abdel-Khalek AA (2015). Antioxidant responses and nuclear deformations in freshwater fish, O*reochromis niloticus*, facing degraded environmental conditions. Bull Environ Contam Toxicol.

[CR2] Abdel-Khalek AA, Elhaddad E, Mamdouh S, Marie MAS (2018). The chronic exposure to discharges of Sabal drain induces oxidative stress and histopathological alterations in *Oreochromis niloticus*. Bull Environ Contam Toxicol.

[CR3] Abdel-Khalek AA, Zayed HS, Elsayad SM, Zaghloul KH (2020). Assessment of metal pollution impacts on *Tilapia zillii* and *Mugil cephalus* inhabiting Qaroun and Wadi El-Rayan lakes, Egypt, using integrated biomarkers. Environ Sci Pollut Res.

[CR4] Adeogun AO, Ibor OR, Omiwole R, Chukwuka AV, Adewale AH, Kumuyi O, Arukwe A (2020). Sex-differences in physiological and oxidative stress responses and heavy metals burden in the black jaw tilapia, *Sarotherodon melanotheron* from a tropical freshwater dam (Nigeria). Comp Biochem Physiol Part-c: Toxicol Pharmacol.

[CR5] Ali H, Khan E (2018). Bioaccumulation of non-essential hazardous heavy metals and metalloids in freshwater fish. Risk to human health. Environ Chem Lett.

[CR6] Antoine JMR, Fung LAH, Grant CN (2017). Assessment of the potential health risks associated with the aluminium, arsenic, cadmium and lead content in selected fruits and vegetables grown in Jamaica. Toxicol Rep.

[CR7] Barbier O, Jacquillet G, Tauc M, Cougnon M, Poujeol P (2005). Effect of heavy metals on, and handling by, the kidney. Nephron Physiol.

[CR8] Chatterjee N, Walker GC (2017). Mechanisms of DNA damage, repair and mutagenesis. Environ Mol Mutagen.

[CR9] Chaudhuri A, Podder A, Biswas M, Roy A, Homechaudhuri S (2021). Characterization of chloride cells coupled with immunolocalization of Na^+^ K^+^ ATPase in fishes of different migratory guilds of Indian Sundarbans. Reg Stud Mar Sci.

[CR10] da Mota Araujo HR, Fernandes MN, da Cruz AL (2018). Gill morphology and Na^+^ /K^+^-ATPase activity of *Gobionellus oceanicus* (Teleostei: Gobiidae) in an estuarine system. Biol Trace Elem Res.

[CR11] El Agawany N, Kaamoush M, El-Zeiny A, Ahmed M (2021). Effect of heavy metals on protein content of marine unicellular green alga *Dunaliella tertiolecta*. Environ Monit Assess.

[CR12] Elwasify AYH, Ghanem MH, El-bamby MMM, Ali FAF (2021). Impact of bioaccumulation and biosedimentation of some heavy metals on some biochemical responses in the sole fish, Solea solea inhabiting Lake Qarun, Egypt. Egypt J Aquat Biol Fish.

[CR13] Gercel-Taylor C, Ackermann MA, Taylor DD (2001). Evaluation of cell proliferation and cell death based assays in chemosensitivity testing. Anticancer Res.

[CR14] Hamilton PB, Cowx IG, Oleksiak MF, Griffiths AM, Grahn M, Stevens JR, Carvalho GR, Nicol E, Tyler CR (2016). Population-level consequences for wild fish exposed to sublethal concentrations of chemicals – a critical review. Fish Fish (oxf).

[CR15] Javed M, Usmani N (2019). An overview of the adverse effects of heavy metal contamination on fish health. Proc Natl Acad Sci India Sect B Biol Sci.

[CR16] Javed M, Ahmad I, Usmani N, Ahmad M (2016). Studies on biomarkers of oxidative stress and associated genotoxicity and histopathology in *Channa punctatus* from heavy metal polluted canal. Chemosphere.

[CR17] Jindal R, Verma S (2015). In vivo genotoxicity and cytotoxicity assessment of cadmium chloride in peripheral erythrocytes of *Labeo rohita* (Hamilton). Ecotoxicol Environ Saf.

[CR18] Khallaf EA, Authman MMN, Alne-na-ei AA (2018). Contamination and Ecological Hazard Assessment of Heavy Metals in Freshwater Sediments and *Oreochromis niloticus* (Linnaeus, 1758) Fish Muscles in a Nile River Canal in Egypt. Environ Sci Pollut Res.

[CR19] Kontaş S, Bostancı D (2020). Genotoxic effects of environmental pollutant heavy metals on Alburnus chalcoides (Pisces: Cyprinidae) inhabiting lower Melet River (Ordu, Turkey). Bull Environ Contam Toxicol.

[CR20] Lall SP, Kaushik SJ (2021). Nutrition and metabolism of minerals in fish. Animals.

[CR21] MacDonald DD, Ingersoll CG, Berger TA (2000). Development and evaluation of consensus-based sediment quality guidelines for freshwater ecosystems. Arch Environ Contam Toxicol.

[CR22] MWRI (Ministry of Water Resources and Irrigation) (2013) National Water Resources Plan/Planning Sector. Country Strategy, Egypt. Ministerial Decree No. 92 of 2013 amending the ministerial decree No. 8 of 1982 on the executive regulations of law No. 48 of 1982 concerning the protection of the Nile River and water channels from pollution

[CR23] Nagamatsu PC, Vargas DÁR, Prodocimo MM, Opuskevitch I, Ferreira FCAS, Zanchin N, de Oliveira Ribeiro CA, de Souza C (2021). Synthetic fish metallothionein design as a potential tool for monitoring toxic metals in water. Environ Sci Pollut Res.

[CR24] Neugebauer EA, Cartier GLS, Wakeford BJ (2000) Methods for the determination of metals in wildlife tissues using various atomic absorption spectrophotometry techniques. Can. Wildl. Serv. (Issue 337)

[CR25] Pal D, Maiti SK (2018). Seasonal variation of heavy metals in water, sediment, and highly consumed cultured fish (*Labeo rohita* and *Labeo bata*) and potential health risk assessment in aquaculture pond of the coal city, Dhanbad (India). Environ Sci Pollut Res.

[CR26] Paul S, Mandal A, Bhattacharjee P, Chakraborty S, Paul R, Kumar Mukhopadhyay B (2019). Evaluation of water quality and toxicity after exposure of lead nitrate in fresh water fish, major source of water pollution. Egypt J Aquat Res.

[CR27] Pellegri V, Gorbi G, Buschini A (2020). DNA damage detection by Comet Assay on *Daphnia magna*: application in freshwater biomonitoring. Sci Total Environ.

[CR28] Rajeshkumar S, Liu Y, Zhang X, Ravikumar B, Bai G, Li X (2018). Studies on seasonal pollution of heavy metals in water, sediment, fish and oyster from the Meiliang Bay of Taihu Lake in China. Chemosphere.

[CR29] Ratn A, Prasad R, Awasthi Y, Kumar M, Misra A, Trivedi SP (2018). Zn2+ induced molecular responses associated with oxidative stress, DNA damage and histopathological lesions in liver and kidney of the fish, Channa punctatus (Bloch, 1793). Ecotoxicol Environ Saf.

[CR30] Recio L, Hobbs C, Caspary W, Witt KL (2010). Dose-response assessment of four genotoxic chemicals in a combined mouse and rat micronucleus (MN) and Comet assay protocol. Toxicol Sci.

[CR31] Redwan M, Elhaddad E (2017). Heavy metals seasonal variability and distribution in Lake Qaroun sediments, El-Fayoum. Egypt J African Earth Sci.

[CR32] Russell A, Macfarlane GR, Nowak B, Moltschaniwskyj NA, Taylor MD (2019). Lethal and sub-lethal effects of aluminium on a juvenile penaeid shrimp. Thalassas.

[CR33] Samuel MS, Datta S, Khandge RS, Selvarajan E (2021). A state of the art review on characterization of heavy metal binding metallothioneins proteins and their widespread applications. Sci Total Environ.

[CR34] Shah N, Khan A, Habib Khan N, Khisroon M (2021). Genotoxic consequences in common grass carp (*Ctenopharyngodon idella Valenciennes*, 1844) exposed to selected toxic metals. Biol Trace Elem Res.

[CR35] Tice RR, Agurell E, Anderson D, Burlinson B, Hartmann A, Kobayashi H, Miyamae Y, Rojas E, Ryu JC, Sasaki YF (2000). Single cell gel/comet assay: guidelines for in vitro and in vivo genetic toxicology. Environ Mol Mutagen.

[CR36] Tunçsoy M, Duran S, Ay Ö, Cicik B, Erdem C (2017). Effects of copper oxide nanoparticles on antioxidant enzyme activities and on tissue accumulation of *Oreochromis niloticus*. Bull Environ Contam Toxicol.

[CR37] USEPA (2000) Guidance for assessing chemical contaminant data for use in fish advisories. Vol 2, Risk assessment and fish consumption limits (3rd ed.), United States Environmental Protection Agency, Washington, DC, Office of Science and Technology and Office of Water, (EPA/823/B-97/009)

[CR38] USEPA (2012) Integrated Risk Information System (IRIS) of the US Environmental Protection Agency

[CR39] Usero J, González-Regalado E, Gracia I (1997). Trace metals in the bivalve molluscs *Ruditapes decussatus* and *Ruditapes philippinarum* from the Atlantic Coast of Southern Spain. Environ Int.

[CR40] Waseem A, Arshad J (2016). A review of human biomonitoring studies of trace elements in Pakistan. Chemosphere.

[CR41] Xu Z, Liu J, Wang E, Zhao C, Hu X, Chu KH, Wang L (2021). Detoxification and recovery after cadmium exposure in the freshwater crab *Sinopotamon henanense*. Environ Sci Pollut Res.

[CR42] Zaghloul KH, Omar WA, Abdel-Khalek AA, Abo-Hegab S (2011). Ecological monitoring of Mediterranean *Solea aegyptiaca* transplanted into Lake Qaroun. Egypt Aust J Basic Appl Sci.

